# Examining the differential use of a North American animal poison control call center by veterinarians and the public for dog-related calls

**DOI:** 10.1371/journal.pone.0276959

**Published:** 2022-11-16

**Authors:** Keana Shahin, David L. Pearl, Carolyn Martinko, Olaf Berke, Terri L. O’Sullivan

**Affiliations:** Department of Population Medicine, Ontario Veterinary College, University of Guelph, Guelph, ON, Canada; Universidade Federal de Minas Gerais, BRAZIL

## Abstract

Tele-triage, a subset of telehealth services, is becoming increasingly common, they offer users the ability to receive credible health advice from licensed professionals in the comfort of their own home. In the field of veterinary medicine, tele-triage services have been employed since the early 2000s, but there has been little examination of how these services are used by callers. The objectives of this study were to explore how the use of an animal poison control center (APCC) tele-triage service varied between veterinarians and the public in terms of toxicant type, animal demographics, availability of veterinary services, as well as seasonal and secular trends. Data regarding dog poisoning events were obtained from the APCC of the American Society for the Prevention of Cruelty to Animals’ (ASPCA). We fitted a mixed logistic regression model with random intercepts for county and state and identified associations between caller type and the following: animal characteristics (i.e., age, weight, breed-class), type of toxicant, season, year, and access to veterinary services (i.e., veterinarians per capita in the county of the caller). The model included interaction effects between season and both plant and pesticide toxicants. There was also an interaction between year and access to veterinary care. Further investigations are needed to understand how the novelty of a toxicant and the severity of clinical signs associated with a toxicant predict the type of caller, if pet demographics are associated with the caller based on medical issues or owner attitudes, and how access to veterinary care influences the use of this tele-triage service.

## Introduction

In the past decade, there have been significant technological advancements resulting in quicker and easier access to healthcare [1]. Among these advancements, tele-triage services have proven useful since they provide a means of delivering healthcare through communication technology without geographical barriers [1]. Tele-triage refers to the assessment, advice, and intervention for health issues by telephone to clients from a healthcare worker [2]. The use of tele-triage in the veterinary field has become a growing topic of interest [3]. The American Veterinary Medical Association defines veterinary tele-triage as an “umbrella term” for the use of technology to deliver healthcare data, education, and services remotely [3]. Veterinary medicine has been evolving alongside human medicine, and the first use of a veterinary tele-triage service was in New York in the 1980s where a trans-telephonic electrocardiogram transmitter was used to connect veterinarians across America [4]. There are presently a variety of tele-triage options for pet owners to use for their pets in North America [5]. Tele-triage services are very attractive to clients since they are easily accessible, lessen travel time to a clinic, are often cheaper, relieve areas with shortages of veterinarians, and increase access to specialists [4]. One field in veterinary medicine that uses tele-triage services to support clients is veterinary toxicology. Accidental poisonings in pets occur regularly, often due to household toxicants found inside and near the home [6, 7]. Emergency veterinary clinicians often find themselves caring for a client exposed to a potentially toxic substance [6]. Dogs ingest plants, foods, and human and veterinary medicines that can be potentially life threatening; the improper handling of these toxicants by humans often leads to the ingestion of these substances by pets, livestock, and wildlife [8, 9].

The Animal Poison Control Center (APCC) is a 24-hour emergency poison control hotline administered by the American Society for the Prevention of Cruelty to Animals (ASPCA) [8]. The APCC is a tele-triage service that provides callers with emergency toxicological advice to help care for an animal that has potentially been exposed to a poisonous substance. To access services from the APCC, callers were charged 65 USD during the study period, since then the fee has increased to 95 USD; the service is available to the public, veterinarians, and other poison control centers [8]. The APCC receives calls from across the United States of America (US) and Canada, as well as US commonwealths and territories [8]. When a call is received, staff at the APCC collect data about the number of animals exposed, patient characteristics, clinical signs at time of call, outcome, source of report, geographic location of report, and time of call [8].

While the practicality of tele-triage services has been established, there is little research regarding the use of veterinary tele-triage services. To better inform policy, and to improve the system’s performance, it is imperative to determine why and how various stakeholder groups access tele-triage services. Our major hypotheses were that the probability of a call for a dog being from the general public compared to veterinarians will be higher in communities with less access to veterinary services, those involving more common toxicants, and that dog characteristics (e.g., breed and age) would influence the type of caller. We also anticipated that the effect of access to veterinary care would change over secular time, and seasonal time would modify the relationships between environmental toxicant types (i.e., plant toxicants and pesticides) and the type of caller.

Consequently, this study’s objectives were to explore how the use of the APCC varies between veterinarians and the public in terms of the following characteristics: type of toxicant, dog demographics (age, breed type, sex, reproductive status), availability of veterinary services, and seasonal and secular time.

## Materials and methods

### Data

For this study, we received data on poisoning events and dog-level demographics of the animals involved from the ASPCA’s APCC from 2005 to 2014. As the data are secondary and there were no interactions between animals, the researchers, or the staff that collected the original call data, approval by an animal research ethics committee was not required. During each call, the APCC collects data concerning the number of animals affected, clinical effects, patient outcome, toxicant information, as well as the date/time/location of the phone call. The APCC stores this information in their AnTox toxicological database.

For this study, an observation included data for each dog that was exposed to a potential toxicant (e.g., food, plant, pesticide, human medication, veterinary medication, or illicit/recreational drug) that was recorded during a call to the APCC [8]. Route of exposure was not considered in this study. The data used in this study from the AnTox database included 179,724 unique events. The dog-level variables of interest from the AnTox database were weight (kilograms), sex, age, reproductive status, breed class, type of toxicant, season, and call source. The toxicant categories accounted for more than one type of substance; the food category for instance accounted for poisoning events from a variety of foods including: alcohol, avocado (*Persea americana)*, caffeine, chocolate (*Theobroma cacao* derived product*)*, citrus fruits (*Citrum)*, coconut (*Cocos nucifera)*, dairy, grapes (*Vitis vinifera)*, nuts, onion (*Allium cepa)*, garlic (*Allium sativum)*, chives (*Allium schoenoprasum*), and xylitol (natural sugar substitute). The human medicine category included analgesics (e.g., acetaminophen), antihistamines (e.g., diphenhydramine), cardiovascular drugs (e.g., enalapril), central nervous system drugs (e.g., diazepam), gastrointestinal drugs (e.g., prucalopride), respiratory drugs (e.g., albuterol), birth control/contraceptives (e.g., levonorgestrel), endocrine drugs (e.g., pasireotide), dermatological drugs (e.g., topical betamethasone), cancer medications (e.g., tamoxifen), musculoskeletal drugs (e.g., carisoprodol), antibiotics (e.g., amoxicillin), antivirals (e.g., peginterferon), antifungals (e.g., miconazole), anti-parasitic (e.g., miltefosine), ear/nose/throat drugs (e.g., Ciprodex–an otic formulation of ciprofloxacin and dexamethasone to treat ear infections), and immunosuppressants (e.g., prednisone). Within the veterinary medicine category, we included certain anti-parasitic (e.g., eprinomectin), analgesics (e.g., meloxicam), urinary incontinence drugs (e.g., phenylpropanolamine), vaccines (e.g., various rabies vaccines), antibiotics (e.g., florfenicol), antifungals (e.g., miconazole), and central nervous system drugs (e.g., acepromazine). Medications were classified as human or veterinary drugs based upon their marketed use. For example, if an antibiotic was marketed to treat human acne it would be included as a human drug. The illicit/recreational drugs variable consisted of tobacco products, cannabis, and illegal substances such as heroin and methamphetamine. An in-depth list of toxicants within the above categories used for our analyses is provided by Swirski et al. [8].

The database also included each dog’s primary breed. This information was used to categorize the dogs into the American Kennel Club (AKC) breed classes: herding, hound, non-sporting, sporting, terrier, toy, working, Foundation Stock Service (FSS), and other. Dogs that met the criteria for the AKC’s miscellaneous category were put into the FSS group. Some observations for age and weight were recorded as unlikely values and were therefore treated as missing data. Ages recorded as “0” (n = 831) or greater than 26 years old (n = 9) were not used in this study. Weights recorded as “0” (n = 812) or exceeding 114 kg for giant breed dogs (Great Danes, Mastiffs, Neapolitan Mastiffs, Tibetan Mastiffs, Leonbergers, Boerboels, Newfoundlands, St. Bernard’s) (n = 0) or exceeding 75 kg for all other breeds were not used in this study (n = 17). The original coding in the AnTox database for the reproductive status variable was immature, neutered, intact, pregnant, lactating, or unknown. This coding was collapsed for subsequent analyses into the following categories: intact (included pregnant, lactating, and immature), neutered, or unknown. The sex variable was originally coded in the AnTox database as female, male, did not ask, group, and unknown, and was recoded as female, male, or unknown, for subsequent analyses. The season variable was coded as winter (December, January, February), fall (September, October, November), spring (March, April, May), and summer (June, July, August).

The source of the call to the APCC was logged in the AnTox database as veterinarian, public, other poison control center (n = 7), and Animal Product Safety Service (n = 36). For this study, only calls from veterinarians and the public were analyzed. Dogs were linked to the counties where their call originated using the location information collected by the APCC; 2010 US census data were used to estimate the number of veterinarians per 100,000 population in each county.

### Statistical analyses

Descriptive statistics were reported; these included means, medians, standard deviations, and 95% confidence intervals. The descriptive statistics reported were based on the type of data (i.e., nominal, ordinal, or continuous). The correlation between independent variables was examined using various correlation coefficients (e.g., Phi coefficient, Spearman rank correlation coefficient) depending on the type of independent variables. If the correlation between two variables was greater than |0.75|, the more epidemiologically plausible variable was kept in the model for subsequent multivariable modelling to avoid issues with collinearity. Linearity was assessed between each continuous independent variable and the log odds of the outcome using locally weighted linear regression (LOWESS) and by assessing the statistical significance of adding a quadratic term. If the relationship was not linear, the independent variable was categorized or modeled as a quadratic relationship if appropriate.

Mixed effects univariable logistic regression models were fitted to assess the associations between source of call and the following independent variables: toxin type (i.e., season, plants, pesticides, human medicine, veterinary medicine, food, illicit/recreational drugs), weight, age, year, number of veterinarians per 100,000 people in the county, and sex. Random intercepts for county and state were included in univariable models and the final multivariable model to account for clustering by county and state. In multi-dog household poisonings, we randomly selected one dog from each household to avoid issues with model convergence when we attempted to include a random intercept for household. Independent variables with significant associations were considered for inclusion in the final multivariable model. Using a manual backward elimination process, variables were included in the final multivariable model if they were statistically significant, were part of a significant interaction term, or acted as distorter variables or explanatory antecedents (i.e., confounders). Distorter variables and explanatory antecedents included non-intervening variables whose removal resulted in a 20% or greater change in the coefficient of a statistically significant variable. We examined interactions between veterinarians per 100,000 people in the county and year, as well as season and plants and season and pesticides. A significance level of 5% (i.e., alpha = 0.05) was used for all univariable and multivariable models. For categorical variables with more than 2 categories, a Wald’s chi-squared test was used to determine the significance of the entire variable. Contrasts were performed to examine statistically significant interaction effects involving categorical variables and graphs of the predicted log odds were used to examine interactions involving continuous variables. The best linear unbiased predictions (BLUPs) were examined graphically to assess that the BLUPs met the assumptions of homoscedasticity and normality, and Pearson residuals were used to identify outliers. Variance partition coefficients were estimated using the latent variable technique [10]. All statistical analyses were performed using STATA 16 (StataCorp, College Station, TX). Due to concerns over the misapplication of the term “statistically significant” [11], we highlight that the term does not imply causation or biological importance. We use the term “statistically significant” in an exploratory sense [12].

## Results

### Descriptive statistics

**Fig 1:**
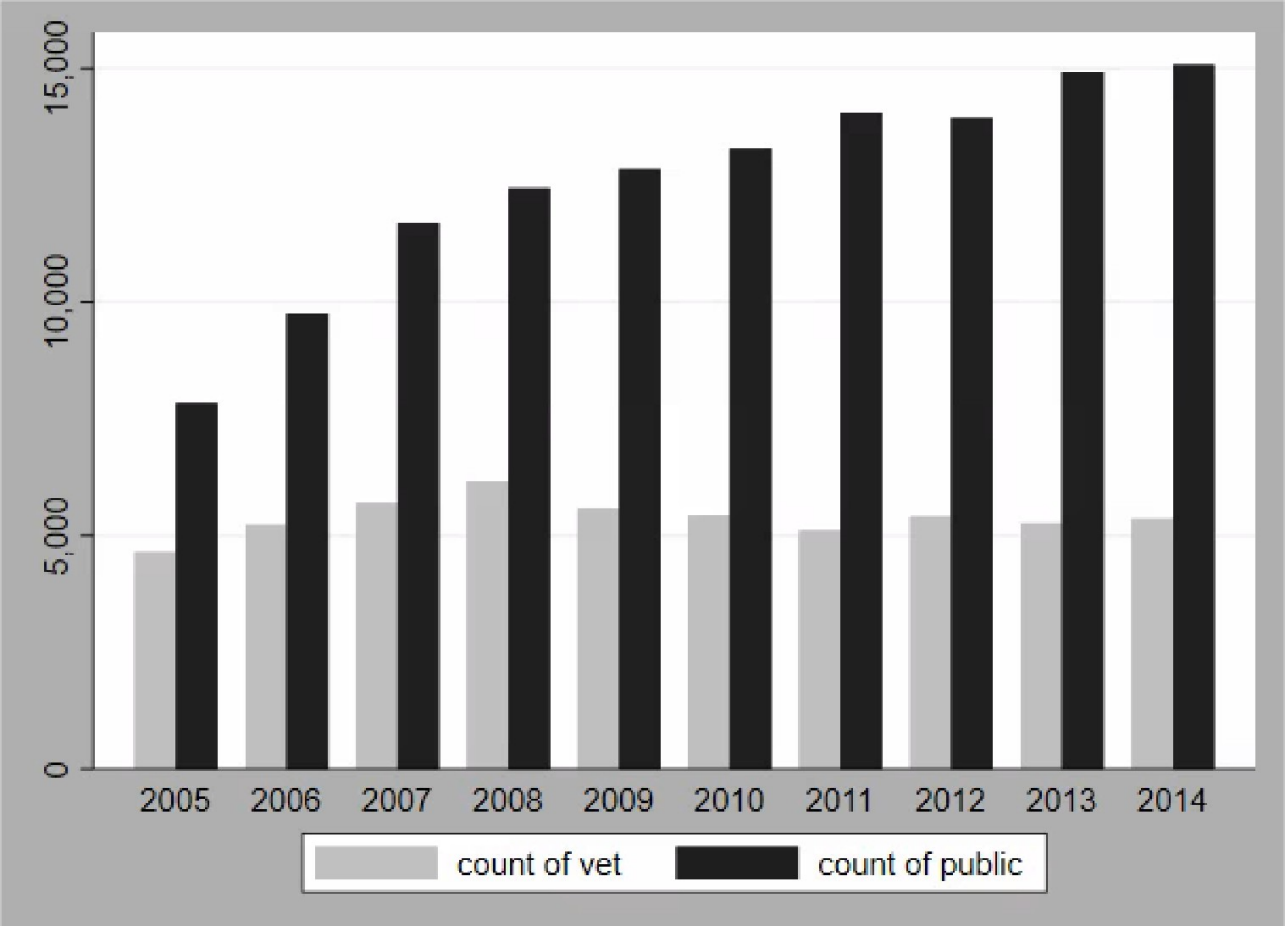
Total calls to the APCC by caller type (veterinarian & public) and year the call was received (2005–2014).

More calls to the APCC were made by dog owners than by veterinarians during the study period (2005–2014) (Table 1, Fig 1). The number of calls by veterinarians remained relatively constant over the study period, while the number of calls by the public increased between 2005–2014 (Fig 1). There were more calls concerning female dogs than male dogs (Table 1). In terms of breed class, the toy and sporting groups made up the largest proportion of calls (Table 1). There were more calls from owners of neutered/spayed dogs than intact dogs (Table 1). The average weight of a dog in this study was 16.4 kg (SD = 12.6) and the average age was 3.7 years old (SD = 3.5). The density of veterinarians per 100,000 population ranged from 2.29 to 162.45 among counties where calls originated. The most common type of toxicant was human medication (Table 1).

**Table 1:** Descriptive statistics concerning the source of calls and the characteristics of the dogs, availability of veterinary services, type of toxicants, and season from calls made to the APCC concerning poisoning events in pet dogs from the US (2005–2014)*.

Variable	Total	Percentage of dataset
**Call source**		
Public	125,818	70.01
Veterinarian	53,906	29.29
**Breed class**		
Herd	15,130	8.42
Hound	16,162	8.99
Foundation Stock Services	491	0.27
Non-Sporting	15,406	8.57
Sporting	41,982	23.36
Terrier	19,555	10.88
Toy	43,918	24.44
Working	15,625	8.69
Other	11,456	6.37
**Reproductive status**		
Intact	39,839	22.17
Neutered	139,885	77.83
**Sex**		
Male	86,420	48.08
Female	93,304	51.92
**Age quartile** ^**b**^		
1^st^ Quartile (0.1 year– 0.9 year)	46,569	25.91
2^nd^ Quartile (1 year– 2 years)	44,145	24.56
3^rd^ Quartile (2.1 years– 6 years)	51,004	28.38
4^th^ Quartile (6.1 years– 23 years)	38,006	21.15
**Veterinary medicine**		
No	168,422	93.71
Yes	11,302	6.29
**Human medicine**		
No	123,052	68.47
Yes	56,672	31.53
**Plants**		
No	148,710	82.74
Yes	15,629	8.70
**Food**		
No	148,710	82.74
Yes	31,014	17.26
**Cleaning products**		
No	171,356	95.34
Yes	8,368	4.66
**Pesticides**		
No	154,610	86.03
Yes	25,114	13.97
**Dietary supplements**		
No	166,338	92.55
Yes	13,386	7.45
**Illicit/recreational drugs**		
No	176,451	98.18
Yes	3,273	1.82
**Season**		
Winter	43,684	25.43
Summer	46,523	25.89
Fall	43,815	24.38
Spring	45,704	24.31
**Quantiles**^**b**^ **of vets per 100,000 people**		
1^st^ Quantile (2.29–16.60 per 100,000)	59,799	33.39
2^nd^ Quantile (16.69–25.74 per 100,000)	59,966	33.49
3^rd^ Quantile (25.96–162.45 per 100,000)	59,317	33.12

*n = 179,274 for all variables except for quantiles of vets per 100,000 people where n = 179,082.

^b^ Ranges for quantiles/quartiles represent actual measurements within the interval.

### Mixed univariable logistic regression models

The results of the mixed effects univariable logistic regression models indicated that the following variables were significantly associated with the source of a call: reproductive status, breed class, weight, age, human medicine, veterinary medicine, illicit/recreational drugs, food, dietary supplements, plant toxicants, pesticides, cleaning products, season, and year (Table 2).

**Table 2:** Results of *mixed effects univariable logistic regression concerning the association between source of calls (1 = Veterinarian, 0 = Public) to the APCC (2005–2014) and dog demographics, type of toxicant, season, year, and availability of veterinary services.

Variable	Odds Ratio	95% Confidence Int	P-Value
**Breed class**			
Herding	Referent		
Hound	0.98	0.93–1.03	0.395
Foundation stock services	0.79	0.64–0.98	**0.029**
Non-Sporting	1.04	0.99–1.09	0.135
Sporting	0.93	0.89–0.97	**<0.001**
Terrier	1.05	1.003–1.104	**0.036**
Toy	1.09	1.05–1.14	**<0.001**
Working	0.95	0.91–1.00	0.061
Other	1.24	1.18–1.31	**<0.001**
**Reproductive status**			
Intact	Referent		
Neutered	0.77	0.75–0.79	**<0.001**
**Sex**			
Male	Referent		
Female	1.01	0.99–1.04	0.301
**Age quartiles** ^**a**^			
1st Quartile (0.1 year– 0.9 year)	Referent		
2nd Quartile (1 year– 2 years)	0.97	0.95–1.00	0.073
3rd Quartile (2.1 years– 6 years)	0.88	0.86–0.91	**<0.001**
4th Quartile (6.1 years– 23 years)	0.97	0.938–0.997	**0.033**
**Weight**	0.995	0.994–0.996	**<0.001**
**Veterinary medicine**			
No	Referent		
Yes	1.63	1.56–1.69	**<0.001**
**Human medicine**			
No	Referent		
Yes	1.27	1.25–1.30	**<0.001**
**Plants**			
No	Referent		
Yes	0.91	0.88–0.95	**<0.001**
**Food**			
No	Referent		
Yes	0.71	0.69–0.74	**<0.001**
**Cleaning products**			
No	Referent		
Yes	0.93	0.89–0.98	**0.005**
**Pesticides**			
No	Referent		
Yes	1.24	1.20–1.27	**<0.001**
**Dietary supplements**			
No	Referent		
Yes	0.75	0.72–0.78	**<0.001**
**Illicit/recreational Drugs**			
No	Referent		
Yes	1.55	1.44–1.66	**<0.001**
**Season**			
Winter	Referent		
Summer	1.02	0.99–1.05	0.229
Fall	1.06	1.03–1.09	**<0.001**
Spring	1.04	1.01–1.07	**<0.001**
**Quantiles of vets per 100,000 People** ^**a**^^,^ ^**b**^			
1st Quantile (2.29–16.6 per 100,000)	Referent		
2nd Quantile (16.69–25.74 per 100,000)	1.02	0.89–1.17	0.759
3rd Quantile (25.96–162.45 per 100,000)	1.12	0.98–1.27	0.092
**Year**	0.93	0.93–0.94	**<0.001**

* Random intercept included in each model for county and state.

^a^ Ranges for quantiles/quartiles represent actual measurements.

^b^ This is based on the number of veterinarians per 100,000 people in the county where the event occurred.

### Multivariable mixed logistic regression model

The final multivariable mixed logistic regression model included the following variables and interaction terms: reproductive status, breed class, weight, age, sex, human medicine, veterinary medicine, illicit/recreational drugs, dietary supplements, plant toxicants, pesticides, cleaning products, veterinarians per 100,000 population, season, year, and interactions between season and plant toxicants, season and pesticides, and year and veterinarians per 100,000 population (Table 3).

**Table 3:** Results of mixed effects multivariable logistic regression concerning the associations between source of call (1 = Veterinarian, 0 = Public) to the APCC (2005–2014) and dog demographics, type of toxicant, season, year, and access to veterinary services.

Variable	Odds Ratio	95% Confidence Int	P-Value
**Breed class**			
Herding	Referent		
Hound	0.97	0.92–1.02	0.264
Foundation stock services	0.84	0.68–1.05	0.121
Non-Sporting	1.01	0.96–1.07	0.593
Sporting	0.95	0.91–0.99	**0.011**
Terrier	1.02	0.97–1.07	0.439
Toy	1.02	0.98–1.08	0.261
Working	0.97	0.92–1.02	0.194
Other	1.26	1.19–1.33	**<0.001**
**Reproductive status**			
Intact	Referent		
Neutered	0.77	0.74–0.79	**<0.001**
**Sex**			
Male	Referent		
Female	1.01	0.99–1.03	0.378
**Age quartiles** ^**a**^			
1^st^ Quartile (0.1 year– 0.9 year) ^b^	Referent		
2^nd^ Quartile (1 year– 2 years)	1.13	1.09–1.16	**<0.001**
3^rd^ Quartile (2.1 years– 6 years)	1.10	1.07–1.14	**<0.001**
4^th^ Quartile (6.1 years– 23 years)	1.23	1.19–1.27	**<0.001**
**Weight**	0.998	0.997–0.999	**<0.001**
**Plants** ^**b**^			
No	Referent		
Yes	1.14	1.04–1.24	**0.004**
**Veterinary medication**			
No	Referent		
Yes	2.01	1.92–2.10	**<0.001**
**Cleaning products**			
No	Referent		
Yes	1.16	1.10–1.22	**<0.001**
**Human medication**			
No	Referent		
Yes	1.49	1.45–1.54	**<0.001**
**Pesticides** ^**b**^			
No	Referent		
Yes	1.61	1.49–1.75	**<0.001**
**Dietary supplements**			
No	Referent		
Yes	0.86	0.82–0.90	**<0.001**
**Illicit/recreational drugs**			
No	Referent		
Yes	1.97	1.82–2.12	**<0.001**
Quantiles of vets per 100,000 people*^,^ ^**a**^^,^ ^**b**^			
1^st^ Quantile (2.29–16.6 per 100,000) ^b^	Referent		
2^nd^ Quantile (16.69–25.74 per 100,000)	1.05	0.91–1.22	0.478
3^rd^ Quantile (25.96–162.45 per 100,000)	1.08	0.94–1.23	0.271
Year x quantiles of vets per 100,000 people*^,^ ^**a**^^,^ ^**b**^			
Year x 2^nd^ quantile (16.69–25.74 per 100,000)	0.99	0.99–1.00	0.258
Year x 3^rd^ quantile (25.96–162.45 per 100,000)	1.01	1.002–1.021	**0.017**
**Year** ^**b**^	0.94	0.93–0.94	**<0.001**
**Season** ^**b**^			
Winter	Referent		
Fall	1.01	0.97–1.04	0.696
Spring	1.02	0.99–1.06	0.230
Summer	0.98	0.95–1.01	0.265
**Season x plants** ^**b**^			
Fall x plants	1.19	1.07–1.34	**0.002**
Spring x plants	0.93	0.83–1.04	0.218
Summer x plants	1.00	0.89–1.12	0.950
**Season x pesticides** ^**b**^			
Fall x pesticides	0.91	0.82–1.01	0.067
Spring x pesticides	0.89	0.81–0.98	**0.021**
Summer x pesticides	0.85	0.78–0.94	**<0.001**
**Random effects**	Variance	95% Confidence Interval	
State	0.11	0.06–0 .20	
County	0.29	0.25–0 .34	

*This is based on the number of veterinarians per 100,000 people in the county where the event occurred.

^a^ Ranges for quantiles/quartiles represent actual measurements.

**Fig 2:**
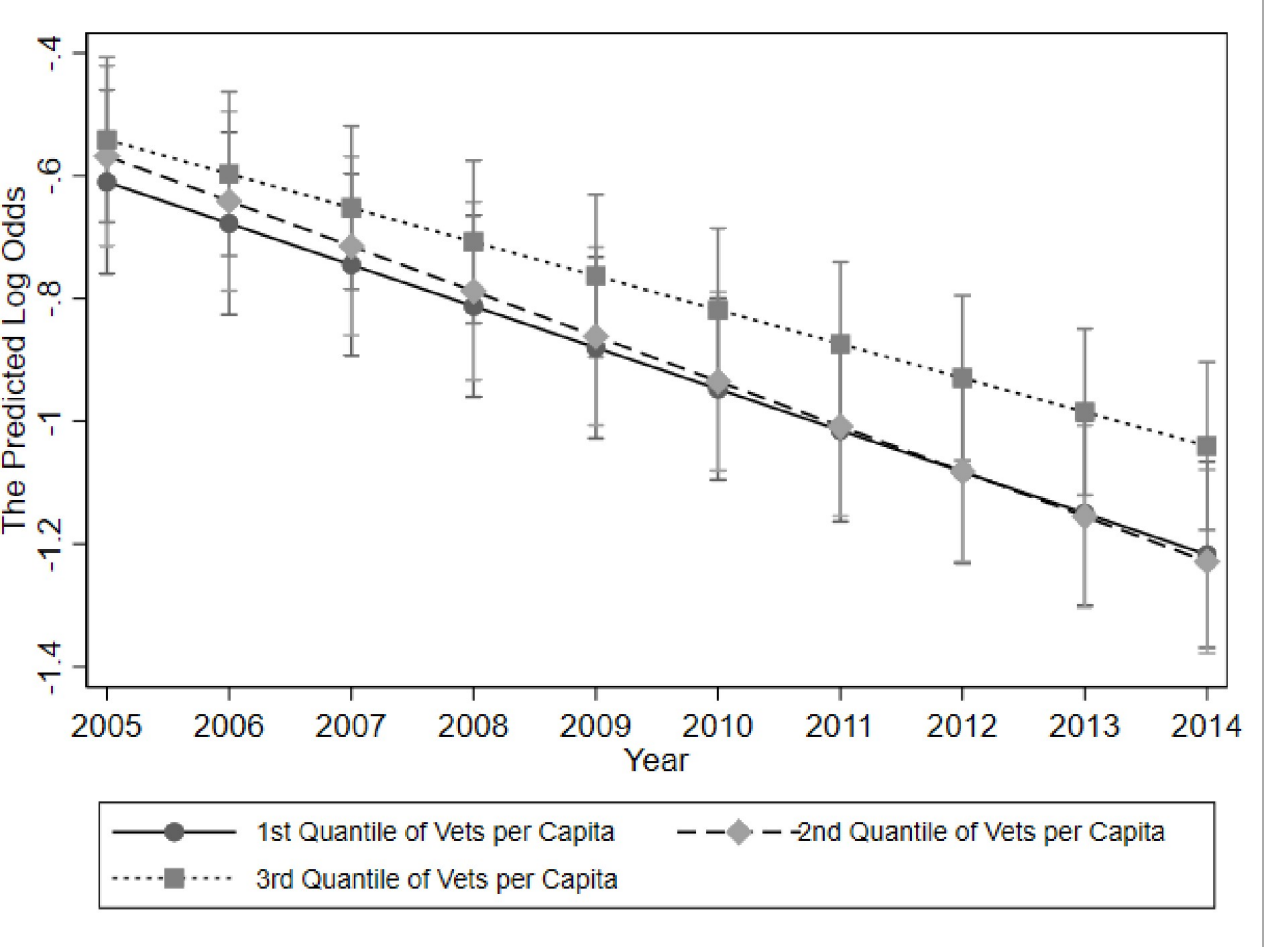
Predicted log odds of a call to the APCC being from a veterinarian from 2005–2014 by quantile of veterinarians per 100,000 population in a county.

^b^ These values refer to exponentiated coefficients; contrasts with the appropriate odds ratios are available in Tables 5 and 6 or predicted outcomes are provided in Fig 2.

#### i. Dog-level variables

Based on the final model, the odds of a call being from a veterinarian were significantly higher for dogs in the higher age quartiles compared to the first quartile (Table 3). The odds of a call being from a veterinarian were significantly lower if the call concerned a neutered dog (Table 3). The odds of a call being from a veterinarian were significantly lower for heavier dogs (Table 3). The odds of a call being from a veterinarian were significantly higher for the other breed group compared to the herding, hound, foundation stock, non-sporting, sporting, terrier, toy, and working breed classes (Table 4). The odds of a call being from a veterinarian were significantly lower in the hound category when compared to the terrier and toy breed groups (Table 4). The odds of a call coming from a veterinarian were significantly higher for the non-sporting breed class when compared to the sporting breed class (Table 4). The odds of a call being from a veterinarian were significantly lower for the sporting breed class when compared to the terrier, toy, and herding breed classes (Table 4). The odds of a call being from a veterinarian were significantly higher when comparing the terrier to the working breed class (Table 4). The odds of a call being from a veterinarian were significantly higher for the toy breed class when compared to the working breed class (Table 4). While sex was not statistically significant, it was retained in the final model since its removal confounded the relationship between breed class and call source.

**Table 4:** Contrast table examining the associations between the breed classes on the odds of a poisoning call to the APCC being from a veterinarian than a pet owner.

Breed Class (Rows are referent)	Herding	Hound	Foundation Stock Service	Non-Sport	Sport	Terrier	Toy	Working	Other
**Herding**	-	-	-	-	-	-	-	-	
**Hound**	OR: 1.03 95%CI: 0.98–1.09	-	-	-	-	-	-	-	-
	P-value: 0.264								
**Foundation stock service**	OR:1.19 95%CI: 0.96–1.47	OR: 1.15 95%CI: 0.93–1.43	-	-	-	-	-	-	-
	P-value:0.121	P-value: 0.201							
**Non-Sport**	OR: 0.99 95%CI: 0.93–1.04	OR: 0.96 95%CI: 0.91–1.01	OR: 0.83 95%CI: 0.67–1.03	-	-	-	-	-	-
	P-value:0.593	P-value: 0.087	P-value:0.093						
**Sport**	**OR: 1.06 95%CI:1.01–1.10**	OR: 1.03 95%CI: 0.98–1.07	OR: 0.89 95%CI:0.72–1.10	**OR: 1.07 95%CI: 1.02–1.12**	-	-	-	-	-
	**P-value:0.011**	P-value:0.265	P-value:0.293	**P-value:0.003**					
**Terrier**	OR: 0.98 95%CI: 0.93–1.03	**OR: 0.95 95%CI: 0.907–0.998**	OR:0.83 95%CI:0.67–1.02	OR:0.99 95%CI:0.95–1.04	**OR:0.93 95%CI:0.89–0.97**	-	-	-	-
	P-value:0.439	**P-value: 0.043**	P-value:0.083	P-value:0.837	**P-value:<0.001**				
**Toy**	OR: 0.97 95%CI:0.93–1.02	**OR: 0.94 95%CI: 0.91–0.99**	OR: 0.82 95%CI:0.66–1.02	OR: 0.99 95%CI:0.95–1.03	**OR:0.92 95%CI:0.88–0.96**	OR: 0.99 95%CI:0.95–1.03	-	-	-
	P-value:0.261	**P-value: 0.009**	P-value:0.071	P-value:0.565	**P-value:<0.001**	P-value:0.715			
**Working**	OR:1.04 95%CI:0.98–1.09	OR:1.00 95%CI:0.95–1.06	OR:0.87 95%CI:0.70–1.08	OR: 1.05 95%CI:0.99–1.11	OR: 0.98 95%CI: 0.94–1.02	**OR:1.06 95%CI:1.002–1.113**	**OR:1.06 95%CI:1.01–1.12**	-	-
	P-value:0.194	P-value:0.864	P-value:0.218	P-value:0.089	P-value:0.342	**P-value:0.044**	**P-value:0.020**		
**Other**	**OR:0.79 95%CI:0.75–0.84**	**OR:0.77 95%CI: 0.73–0.81**	**OR: 0.67 95%CI:0.54–0.83**	**OR: 0.81 95%CI:0.76–0.85**	**OR:0.75 95%CI:0.72–0.79**	**OR:0.81 95%CI:0.77–0.85**	**OR:0.82 95%CI:0.78–0.86**	**OR:0.77 95%CI:0.72–0.81**	**-**
	**P-value:<0.001**	**P-value:<0.001**	**P-value:<0.001**	**P-value:<0.001**	**P-value:<0.001**	**P-value:<0.001**	**P-value:<0.001**	**P-value:<0.001**	

#### ii. Toxicants

The odds of a call being from a veterinarian were significantly higher if the call concerned veterinary medication, human medication, cleaning products, or illicit/recreational drug poisonings (Table 3). We noted there were significantly lower odds of a call being from a veterinarian rather than a pet owner if the call concerned a dietary supplement poisoning event (Table 3).

Furthermore, we identified that season significantly modified the effects of plant and pesticide toxicants (i.e., interaction effects) (Table 3). The odds of a call being from a veterinarian were significantly higher for poisoning events involving plants in the fall when compared to plant poisoning events in the winter, spring, and summer (Table 5). The odds of a call coming from a veterinarian were significantly higher in the fall, winter, and summer if a plant toxicant was involved when compared to calls without plant toxicants in the corresponding seasons (Table 5).

**Table 5:** Contrasts examining the interactions between season and plant toxicants on the odds of a call to the APCC from a veterinarian compared to a member of the public.

Contrast	OR	95% CI	P-value
Plant & Winter vs. Plant & Summer	1.02	0.92–1.14	0.678
**Plant & Winter vs. Plant & Fall**	**0.83**	**0.75–0.93**	**<0.001**
Plant & Winter vs Plant & Spring	1.05	0.94–1.17	0.363
**Plant & Summer vs Plant & Fall**	**0.81**	**0.74–0.90**	**<0.001**
Plant & Summer vs Plant & Spring	1.03	0.93–1.14	0.592
**Plant & Fall vs Plant & Spring**	**1.26**	**1.15–1.40**	**<0.001**
**Plant & Winter vs. Non-plant &Winter**	**1.14**	**1.04–1.24**	**0.004**
**Plant & Summer vs. Non-plant & Summer**	**1.13**	**1.05–1.22**	**<0.001**
**Plant & Fall vs Non-plant & Fall**	**1.36**	**1.26–1.46**	**<0.001**
Plant & Spring vs Non-plant & Spring	1.06	0.98–1.14	0.136

*Contrasts based on model presented in Table 3.

The odds of a call being from a veterinarian were significantly higher for pesticide poisoning events in the winter when compared to the spring and summer (Table 6). The odds of a call coming from a veterinarian were significantly lower for pesticide poisoning events in the summer when compared to the fall and spring (Table 6). The odds of a call coming from a veterinarian were significantly higher in the spring, summer, fall, and winter if a pesticide was involved when compared to calls without pesticide poisonings in the corresponding seasons (Table 6). The odds of a call being from a veterinarian were significantly lower in the summer when compared to the spring when a plant toxicant or pesticide was not involved (Table 7).

**Table 6:** Contrasts examining the interactions between season and pesticide toxicants on the odds of a call to the APCC from a veterinarian compared to a member of the public.

Contrast	OR	95% CI	P-value
**Pesticide & Winter vs. Pesticide & Summer**	**1.20**	**1.09–1.31**	**<0.001**
Pesticide & Winter vs. Pesticide & Fall	1.09	0.99–1.20	0.072
**Pesticide & Winter vs Pesticide & Spring**	**1.10**	**1.002–1.199**	**0.044**
**Pesticide & Summer vs Pesticide & Fall**	**0.91**	**0.85–0.98**	**0.018**
**Pesticide & Summer vs Pesticide & Spring**	**0.92**	**0.86–0.98**	**0.013**
Pesticide & Fall vs Pesticide & Spring	1.00	0.93–1.08	0.900
**Pesticide & Winter vs Non-pesticide & Winter**	**1.61**	**1.49–1.75**	**<0.001**
**Pesticide & Summer vs Non-pesticide & Summer**	**1.37**	**1.30–1.45**	**<0.001**
**Pesticide & Fall vs Non-pesticide & Fall**	**1.47**	**1.37–1.57**	**<0.001**
**Pesticide & Spring vs Non-pesticide & Spring**	**1.44**	**1.36–1.52**	**<0.001**

*Contrasts based on model presented in Table 3.

**Table 7:** Contrasts examining the effect of each season on the odds of a call to the APCC being from a veterinarian than a pet owner when the call does not involve a plant toxicant or pesticide.

Contrast	OR	95% CI	P-value
Winter vs. Summer	1.02	0.99–1.05	0.265
Winter vs. Fall	0.99	0.96–1.03	0.696
Winter vs Spring	0.98	0.95–1.01	0.230
Summer vs Fall	0.97	0.94–1.01	0.138
**Summer vs Spring**	**0.96**	**0.93–0.99**	**0.023**
Fall vs Spring	0.99	0.95–1.02	0.424

*Contrasts based on model presented in Table 3.

#### iii. Secular time and regional effects

We identified that year significantly impacted the effects of number of veterinarians per capita on caller type (i.e., interaction effects) (Table 3). The odds of a call coming from a veterinarian declined over time, while the rate of decline was slower for dogs living in counties with the highest quantile of veterinarians per 100,000 population (Fig 2). In other words, where more veterinarians were available, the increase in proportion of public calls was slower than areas with fewer veterinarians.

The county and state level random effects accounted for 7.9% and 3.0% of the variance (Table 3).

#### iv.Diagnostics

The BLUPs met the assumptions of homoscedasticity and normality, and there were no outliers.

## Discussion

Tele-triage services such as the APCC are useful tools for veterinarians and pet owners alike. Factors that influence an individual’s decision to call the APCC may include the severity of poisoning event and their knowledge of the toxicant. A study found that the public’s knowledge of the toxicity of common household foods for pets is still quite low [13]. This may influence a pet owner’s decision to call the APCC more than a veterinarian who is educated on the subject [13]. A research study from 2018 found that from a sample of 616 pet owners, only approximately half had some knowledge of potentially harmful household toxicants [14]. We found that the total number of calls to the APCC increased in absolute numbers and relative to veterinary calls over the study period. This suggests increasing interest/awareness of the public in this service. However, in addition to toxicants, we also found that dog characteristics and regional effects also influenced differential use of this service.

### Dog-level variables

When examining the associations between weight and the odds of a call coming from a veterinarian, dogs that were heavier had lower odds of a call being from a veterinarian. This may be explained by pet owners’ perceptions of small dogs being fragile and requiring veterinary attention right away if exposed to a toxicant, as well as the amount of a toxicant needed to cause harm to a dog [15]. The age and weight of a dog may also impact the severity of a toxicant exposure (i.e., issues related to dose) which could play a role in influencing the caller because an acute severe reaction may prompt a pet owner to seek a veterinarian immediately rather than call the APCC. When examining pet age, we found that compared to the youngest quartile, calls concerning dogs in older age categories were more likely to come from veterinarians than the public. Previous work has determined that dogs are more likely to develop health problems as they age [16]. Comorbidities in older dogs can cause the severity of toxicant exposures to increase which could prompt a pet owner to take their dog to a veterinarian rather than calling the APCC. In addition to this, the owner of a mature dog may have an established relationship with a veterinarian, whereas the owner of a puppy may not have established a veterinarian relationship yet, leading them to the APCC in the event of a toxicant exposure.

The odds of a call coming from a veterinarian were significantly lower if the dog was neutered. This finding may reflect differences among dog owners who keep their pets intact for breeding purposes and their differential use of veterinary services or reflect a lack of affordable spay/neuter options for these owners.

In our study, we also found an association between a dog’s breed class and type of caller. The differences in caller by breed class may be due to size differences. For example, toy breeds are smaller than hounds and sporting dogs, and a study suggested that toy breeds may be perceived as more fragile as one possible reason to explain the increased odds of a call to the APCC for cannabis intoxication [15]. They also suggested that the amount of a toxicant required to cause harm to a pet would be less for smaller breed dogs [14]. Our study results were consistent with these hypotheses in that the odds of a call by a veterinarian rather than a member of the public were higher for toy breeds relative to hounds and sporting breeds. The choice of breed may also reflect differences in an owner’s personality; Wells et al. measured neuroticism in owners using a questionnaire and noted that owners of “aggressive” breeds, such as those in the herding and working breed classes, are less neurotic than dog owners of “non-aggressive” breeds, such as Golden and Labrador Retrievers [17]. These differences in owner personality, as they relate to choice of dog breed, could influence the decision to call the APCC directly or seek assistance from a veterinarian who might then call the APCC.

### Toxicants

There were lower odds of a call coming from a veterinarian if the dog had ingested dietary supplements, but the odds were higher for a variety of other toxicants including human and veterinary medications. In our multivariable model, there were no significant differences in the odds of a call coming from a veterinarian compared to a member of the public for food products. It is likely that the differences in the source of call may reflect the severity and/or speed of clinical signs related to the toxicant rather than familiarity as we initially hypothesized. Differences in severity of clinical signs may also explain the interactions we observed between the season variable and plants, as well as season and pesticides. We noted the odds of a call coming from a veterinarian were significantly higher for toxicant events involving plants or pesticides during colder than warmer seasons. This may be attributed to the difference in plants and pesticides a pet dog can be exposed to during these seasons perhaps resulting in greater poisoning severity, resulting in calls from veterinarians to the APCC. The pesticide category includes insecticides and rodenticides, and during colder months some insect and rodent pests may come indoors, which may prompt pet owners to place these substances inside their homes making them easier for dogs to access. These chemicals can also cause severe reactions in pet dogs [18], which may prompt a pet owner to take their dog to a veterinarian directly rather than call the APCC first.

### Secular time and regional effects

At present, there is an issue with access to veterinary care among low socio-economic status and underserved communities, causing worsening animal health and welfare [19]. A recent study identified the five most common barriers to veterinary care as: cost, veterinarian-client communication, cultural/language barriers, lack of client education, and access to a veterinary clinic [19]. Lavallee et al. found that many pet owners residing in metropolitan areas do not have access to their own vehicle and must rely on public transportation, which does not allow pets. Young et al. found that of surveyed pet owners, less than half responded that they would contact their veterinarian in the event of a toxicant exposure, while most stated they would rely on the internet [14]. In terms of APCC use, the odds of a call coming from a veterinarian significantly declined over the study period (2005–2014), but the decline was slowest among calls coming from counties with the greatest number of veterinarians per capita (Fig 2). These results are consistent with owners becoming more aware of tele-triage services [17], like the APCC, but that access to care influences the use of these services. Despite identifying the contextual effect of access to care, we still noted that a portion of the variance in caller type was explained at the county and state levels. This suggests that additional regional factors may explain some of the variation in caller type. There were no data available regarding how pet owners became aware of the APCC; this is an important consideration for future research, and also a potential source of bias if veterinarians or veterinary staff suggested owners contact the APCC over a poisoning incident.

## Conclusion

In summary, owners may be more likely to take their pet to a veterinarian if the toxicant results in more immediate or severe clinical signs. Animal characteristics, such as breed class, reproductive status, age, and weight, were also associated with caller type, and this may reflect the impact of toxicants across these dimensions and/or the attitudes and personality of the owners who prefer certain types of dogs. Access to veterinary care, as measured by the number of veterinarians per capita, also influenced the source of caller, and it appears that over time, communities with the most access to veterinary care were less likely to use the APCC compared to underserviced communities. Given the growing application of healthcare technology in the veterinary field, tele-triage and other telehealth services continue to gain popularity amongst the public and veterinarians. In this instance, use of the APCC showed steady growth among the public, demonstrating the services value to the general population of dog owners. More primary investigations of users of these services in terms of needs and demographics is warranted to improve this service. In addition, studies examining the impact of data source (e.g., owner vs veterinarian) on risk analyses concerning specific toxicants using these data are warranted due to concerns of potential systematic bias.
